# Premature mortality attributable to COVID-19: potential years of life lost in 17 countries around the world, January–August 2020

**DOI:** 10.1186/s12889-021-12377-1

**Published:** 2022-01-09

**Authors:** Maider Pagola Ugarte, Souzana Achilleos, Annalisa Quattrocchi, John Gabel, Ourania Kolokotroni, Constantina Constantinou, Nicoletta Nicolaou, Jose Manuel Rodriguez-Llanes, Qian Huang, Olesia Verstiuk, Nataliia Pidmurniak, Jennifer Wenjing Tao, Bo Burström, Petra Klepac, Ivan Erzen, Mario Chong, Manuel Barron, Terje P. Hagen, Zhanna Kalmatayeva, Kairat Davletov, Inbar Zucker, Zalman Kaufman, Maia Kereselidze, Levan Kandelaki, Nolwenn Le Meur, Lucy Goldsmith, Julia A. Critchley, Maria Angelica Pinilla, Gloria Isabel Jaramillo, Domingos Teixeira, Lara Ferrero Goméz, Jackeline Lobato, Carolina Araújo, Joseph Cuthbertson, Catherine M. Bennett, Antonis Polemitis, Andreas Charalambous, Christiana A. Demetriou, On behalf of the C-MOR consortium

**Affiliations:** 1 University of Nicosia Medical School, Nicosia, Cyprus; 2 Department of Primary Care and Population Health, University of Nicosia Medical School, Nicosia, Cyprus; 3 Department of Basic and Clinical Sciences, University of Nicosia Medical School, Nicosia, Cyprus; 4 European Commission Joint Research Centre, Ispra, Italy; 5 South Carolina Center for Rural and Primary Healthcare, Department of Geography, University of South Carolina, Columbia, USA; 6 Faculty of Medicine 2, Bogomolets National Medical University, Kyiv, Ukraine; 7 Department of Molecular Medicine and Surgery, Karolinska Institutet, Stockholm, Sweden; 8 Department of Global Public Health, Karolinska Institutet, Stockholm, Sweden; 9 Department Communicable Diseases, National Institute of Public Health, Ljubljana, Slovenia; 10 Public Health School, National Institute of Public Health, Ljubljana, Slovenia; 11 Facultad de Ingenieria, Universidad del Pacifico, Lima, Peru; 12 Departamento de Economia, Universidad del Pacifico, Lima, Peru; 13 Department of Health Management and Economics, University of Oslo, Oslo, Norway; 14 Faculty of Medicine, Al Farabi Kazakh National University, Almaty, Kazakhstan; 15 Health Research Institute, Al Farabi Kazakh National University, Almaty, Kazakhstan; 16 Israel Center for Disease Control, Ministry of Health, Ramat Gan, Israel; 17 National Center for Disease Control and Public Health, Tbilisi, Georgia; 18 University of Rennes, EHESP, REPERES - EA 7449, F-35000 Rennes, France; 19 Population Health Research Institute and Institute for Infection and Immunity, St George’s, University of London, London, UK; 20 Population Health Research Institute, St George’s, University of London, London, UK; 21 Faculty of Medicine, Universidad Cooperativa de Colombia, Villavicencio, Colombia; 22 Ministry of Health and Social Security, Praia, Cape Verde; 23 Department of Nature, Life and Environment Sciences, Jean Piaget University of Cape Verde, Praia, Cape Verde; 24 Department of Epidemiology and Biostatistics, Institute of Collective Health (ISC), Fluminense Federal University, Niterói, Brazil; 25 Graduate Public Health Program, Institute of Studies in Collective Health (IESC), Federal University of Rio de Janeiro, Rio de Janeiro, Brazil; 26 Monash University Disaster Resilience Initiative, Monash University, Melbourne, Australia; 27 Institute for Health Transformation, Deakin University, Burwood, Australia; 28 University of Nicosia, Nicosia, Cyprus

**Keywords:** COVID-19, SARS-CoV-2, Disease burden, Potential years of life lost, PYLL, Pandemic

## Abstract

**Background:**

Understanding the impact of the burden of COVID-19 is key to successfully navigating the COVID-19 pandemic. As part of a larger investigation on COVID-19 mortality impact, this study aims to estimate the Potential Years of Life Lost (PYLL) in 17 countries and territories across the world (Australia, Brazil, Cape Verde, Colombia, Cyprus, France, Georgia, Israel, Kazakhstan, Peru, Norway, England & Wales, Scotland, Slovenia, Sweden, Ukraine, and the United States [USA]).

**Methods:**

Age- and sex-specific COVID-19 death numbers from primary national sources were collected by an international research consortium. The study period was established based on the availability of data from the inception of the pandemic to the end of August 2020. The PYLL for each country were computed using 80 years as the maximum life expectancy.

**Results:**

As of August 2020, 442,677 (range: 18–185,083) deaths attributed to COVID-19 were recorded in 17 countries which translated to 4,210,654 (range: 112–1,554,225) PYLL. The average PYLL per death was 8.7 years, with substantial variation ranging from 2.7 years in Australia to 19.3 PYLL in Ukraine. North and South American countries as well as England & Wales, Scotland and Sweden experienced the highest PYLL per 100,000 population; whereas Australia, Slovenia and Georgia experienced the lowest. Overall, males experienced higher PYLL rate and higher PYLL per death than females. In most countries, most of the PYLL were observed for people aged over 60 or 65 years, irrespective of sex. Yet, Brazil, Cape Verde, Colombia, Israel, Peru, Scotland, Ukraine, and the USA concentrated most PYLL in younger age groups.

**Conclusions:**

Our results highlight the role of PYLL as a tool to understand the impact of COVID-19 on demographic groups within and across countries, guiding preventive measures to protect these groups under the ongoing pandemic. Continuous monitoring of PYLL is therefore needed to better understand the burden of COVID-19 in terms of premature mortality.

**Supplementary Information:**

The online version contains supplementary material available at 10.1186/s12889-021-12377-1.

## Background

A new zoonotic disease has been affecting the world since 2020. What was first identified as a local outbreak by the Chinese health authorities in December 2019, was declared a pandemic and a public health emergency of international concern by the World Health Organization (WHO) on March 11, 2020. The novel coronavirus disease was named COVID-19, and by the end of 2020, it had caused over 1.7 million deaths worldwide [1].

To date, studies have evidenced that the case fatality rate of COVID-19 increases with age, primarily affecting individuals over 80 years old [2, 3]. However, COVID-19 not only affects the elderly, but is also a cause of premature mortality [4]. As an alternative to death rates, the Potential Years of Life Lost (PYLL) is an accurate measure of premature mortality [5, 6]. PYLL takes into account the death numbers and the age at which the death occurs, giving more weight to deaths at younger ages and less to deaths at older ages. In this sense, PYLL can give a valid assessment of the COVID-19 mortality impact.

PYLL due to COVID-19 has been previously estimated for several countries worldwide in an attempt to quantify the burden of disease in terms of premature mortality [6–8]. The results of these studies highlight the large mortality impact of COVID-19 in the elderly, and a considerable burden in younger age groups, often among those with vulnerable demographics [7]. However, there is still a gap in published literature on PYLL due to COVID-19, as some countries are over-represented in these studies, whereas smaller countries are often not included. A more targeted analysis is needed to identify the most vulnerable population groups, set priorities, and allocate resources to minimize the COVID-19 mortality burden.

The aim of this study was to provide updated information on the mortality burden of the COVID-19 pandemic in several countries around the world using the PYLL measure.

## Methods

### Data collection

An international consortium (C-MOR) consisting of over 50 institutions across 52 countries and six continents was established to investigate the mortality impact of COVID-19. As part of this large international research project, consortium partners collected data from national primary sources in order to investigate all-cause and COVID-19 mortality during the COVID-19 pandemic. Of these, 17 countries (i.e., Australia, Brazil, Cape Verde, Colombia, Cyprus, France, Georgia, Israel, Kazakhstan, Peru, Norway, England & Wales, Scotland, Slovenia, Sweden, Ukraine, and the United States [USA]) collected and provided age-group and sex specific COVID-19 death numbers, from national primary sources, which were included in this study. The period of investigation was comprised between the inception of the pandemic in each participating country to the end of August 2020 (week 35), with the exception of Kazakhstan, where age- and sex-specific data was available only until the end of week 31, 2020. The information was collected during October–November 2020, which allowed us to account for data cleaning and related reporting delays (ranging from a few days to a few weeks) [8–10].

Countries reported COVID-19 deaths using ISO weeks, Epi weeks, national week, or month as a time unit (Supplementary Table S1). For this study, aggregate numbers to the end of August 2020 (week 35) were used.

COVID-19 deaths were defined differently across the participating countries. Eight countries (Brazil, Colombia, Peru, the USA, Cape Verde, Slovenia, Norway, and Israel) reported them as deaths occurring in persons with COVID-19 irrespective of whether COVID-19 was listed as the primary cause of death on the death certificate; meaning COVID-19 was listed either in the chain of events leading to death (cause of death [COD]) or as a contributing condition. In contrast, other eight countries (Australia, Cyprus, Kazakhstan, England & Wales, Georgia, Scotland, Sweden, and Ukraine) considered as COVID-19 deaths only those deaths where COVID-19 was listed on the chain of causes leading to death (COD) [11]. France reported deaths due to COVID-19 only when they occurred in hospitals and nursing homes. Supplementary Table S1 provides a summary of the data provided by each of the participating countries.

In order to facilitate the comparison of the burden of disease in the different countries, death numbers were also expressed per 100,000 population. Total and sex-specific population estimates for each age group were obtained from the World bank [12], except for the UK nations, for which data from the Office for National Statistics [13] was used, and for Cyprus for which Eurostat data [14] was used to include only the population in the Republic of Cyprus government controlled-area. Population data was based on 2019 estimates.

### PYLL calculation

PYLL were computed starting from the inception of the COVID-19 pandemic in each country, up to the end of August (week 35), 2020. However, in the case of Kazakhstan the PYLL were estimated up to the beginning of August (week 31) due to data availability as described before.

PYLL were computed using the formula described in Romeder and McWhinnie (1977) [15], which provides more conservative estimates than other published methodologies and it focuses on the premature mortality of those who die [5, 16–18]. Nevertheless, in this study 80 years was used as the upper age limit, instead of 70 years, as the mean life expectancy at birth of the countries and territories included in this study was 78.8 and as Mitra et al. (2020) also suggested using 80 years as the upper limit [6].

1 $$PYLL={\sum}_{i=1}^{79}{a}_i\times {d}_i={\sum}_{i=1}^{79}\left(80-i-0.5\right)\times {d}_i\kern0.5em$$

Using this equation (Eq. 1), the remaining years of life are calculated based on the upper age limit of 80 years, where d_*i*_ = number of observed deaths between ages *i* and *i* + 1, *a*_*i*_ = remaining years to live until age 80 when death occurs between ages *i* and *i* + 1, *i* is the mid-point of the age group, and 0.5 is a constant when the mid-point is not a whole number. Due to the choice of 80 years as the upper limit, deaths happening over 80 years of age contribute zero PYLL to the calculation. This methodology also assumes uniform distribution of deaths within age groups.

Countries which reported deaths for age groups that extended further than 80 years (e.g., 75–84) (i.e., Cape Verde, Colombia, Israel, Scotland, Slovenia, Ukraine, and the USA), were interrupted at 79 years (e.g., 75–79) and the demographic distribution of each specific country [19] was used to estimate the percentage of the population in the original age group that would remain in the narrower age group. Then, the number of deaths reported was multiplied by this percentage to estimate the number of deaths in the narrower age group, assuming again uniform distribution of deaths within age groups.

PYLL were calculated per person death and as rates (per 100,000 population) (Eq. 2), for the total population and by sex. PYLL rates were also age-standardized (Eq. 3) [15] using the World (WHO 2000–2025) standard population as the reference population for all countries [20].

2 $$Crude\ PYLL\ rate=\frac{PYLL}{population\ under\ 80\ years}\times \mathrm{100,000}\kern0.5em$$

3 $$Age- adjusted\ PYLL\ rate=\sum_{i=1}^{79}\left( PYLL/{P}_i\right)\times \left({P}_{ir}/{N}_r\right)\kern0.5em \times \mathrm{100,000}\kern0.75em$$

Where, *P*_*i*_ = number of people in the age group *i* in the actual population, *P*_*ir*_ = number of people in the age group *i* in the reference population, and *N*_*r*_ = number of people between ages 1 and 79 in the reference population.

In addition, PYLL estimates were obtained per age group. The age groups used for each country were specified by the age groups used by the national primary source from where data were obtained (Supplementary Table S2). To facilitate age group comparisons, each country’s population was also broken down into three large age groups: below 40 or 45 years, 40–59 or 45–64 years, and over 60 or 65 years. For Cape Verde and Ukraine we modified the initial age groups to facilitate the breakdown into the aforementioned three age groups, in a similar way as explained before for countries with age groups extending the 80 years of age. PYLL estimates were also compared based on the COVID-19 deaths definition used as described before (COD versus COD or a contributing condition). Lastly, PYLL rates (per 100,000 population) were plotted against the excess mortality (estimated as difference in mortality rates per 100,000 population between 2020 observed mortality rate and the average mortality rate between 2015 and 2019) as calculated elsewhere [21].

Deaths with unknown age and/or sex (< 1%) were observed for France and Brazil and contributed zero PYLL to the calculation (Supplementary Table S2).

All figures were produced using R Statistical Software, version 3.6.1 (The R Foundation for Statistical Computing, Vienna, Austria).

## Results

### PYLL attributed to COVID-19

**Fig. 1:**
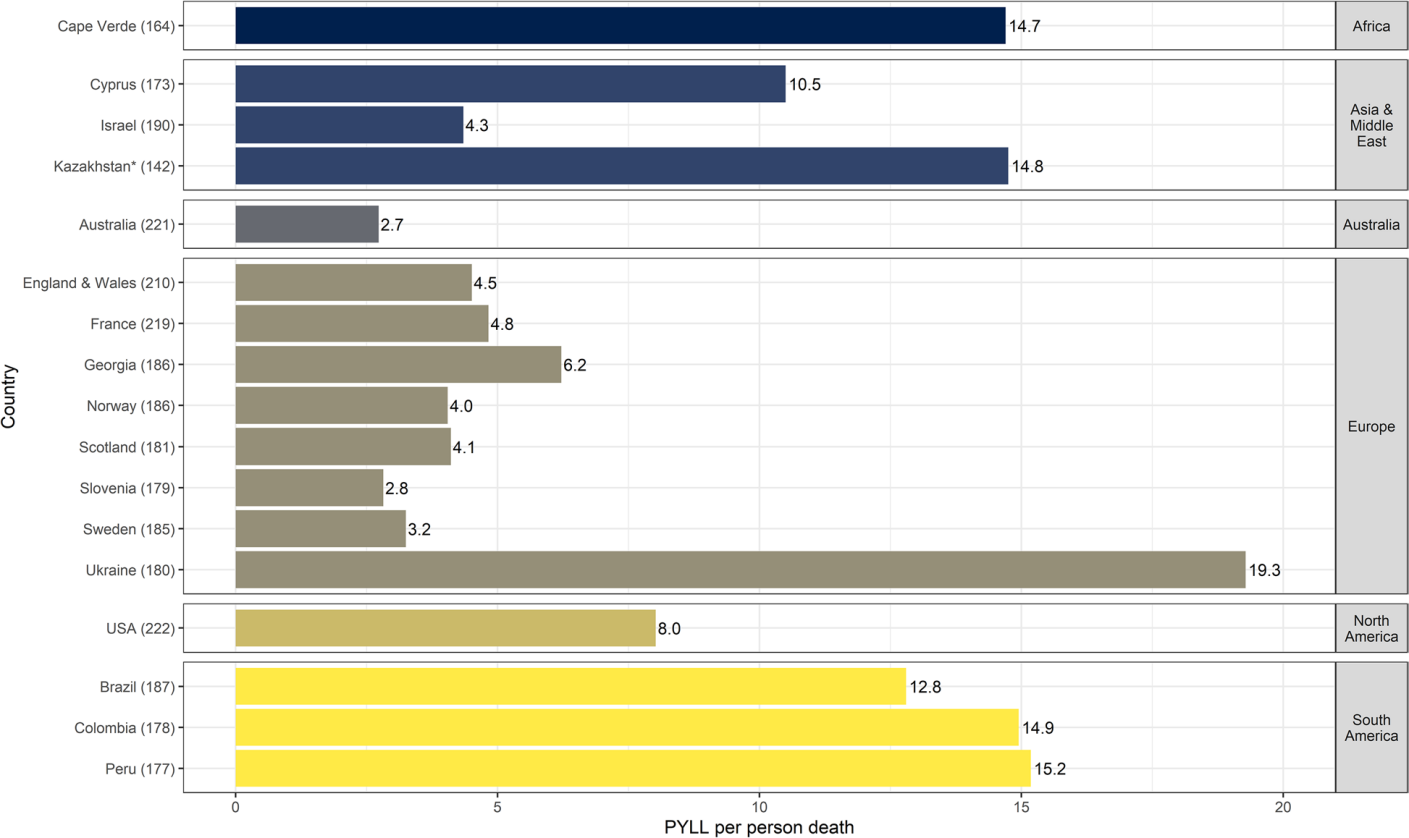
PYLL per person death due to COVID-19 in each of the participating countries up to week 35, 2020. The number in brackets following each country name signifies the number of days from the first COVID-19 case in the country to the last date for which numbers of deaths were available. *For Kazakhstan data was available up to week 31, 2020

As of August 2020, 442,677 (range: 18–185,083) deaths attributed to COVID-19 were recorded in the 17 participating countries, which translated to ~ 4,210,654 (range: 112–1,554,225) PYLL. Figure 1 shows the cumulative PYLL per person death, per country and geographical region, irrespective of COVID-19 death definition used. The average PYLL per person death was 8.7 (range: 2.7–19.3) years. The largest number of PYLL per person death was observed in Ukraine, followed by Peru, Colombia, Kazakhstan, and Cape-Verde. Australia, Israel, and the rest of the European countries, besides Georgia, displayed < 5 PYLL per person death.

**Fig. 2:**
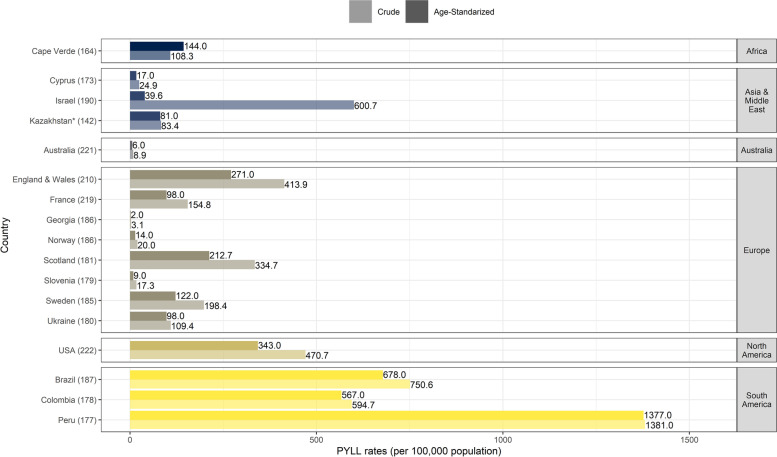
Crude and age-standardized PYLL rates (per 100,000 population) per country up to week 35, 2020. The number in brackets following each country name signifies the number of days from the first COVID-19 case in the country to the last date for which numbers of deaths were available. *For Kazakhstan data was available up to week 31, 2020

South American countries were the sole to experience both the highest PYLL per person and PYLL rates (crude rates ranging from ~ 595 to 1381 PYLL per 100,000 population) (Fig. 2). Across the 17 investigated countries, the crude rates remained below 100 PYLL per 100,000 for Australia, Cyprus, Kazakhstan, Georgia, Norway, and Slovenia. Age-adjusted results followed a similar pattern except for France and Ukraine, where the rates were below 100 PYLL after age adjustment, and Israel in particular whose rate became 15 times lower after being adjusted by age (Fig. 2).

### PYLL attributed to COVID-19 by age groups

**Fig. 3:**
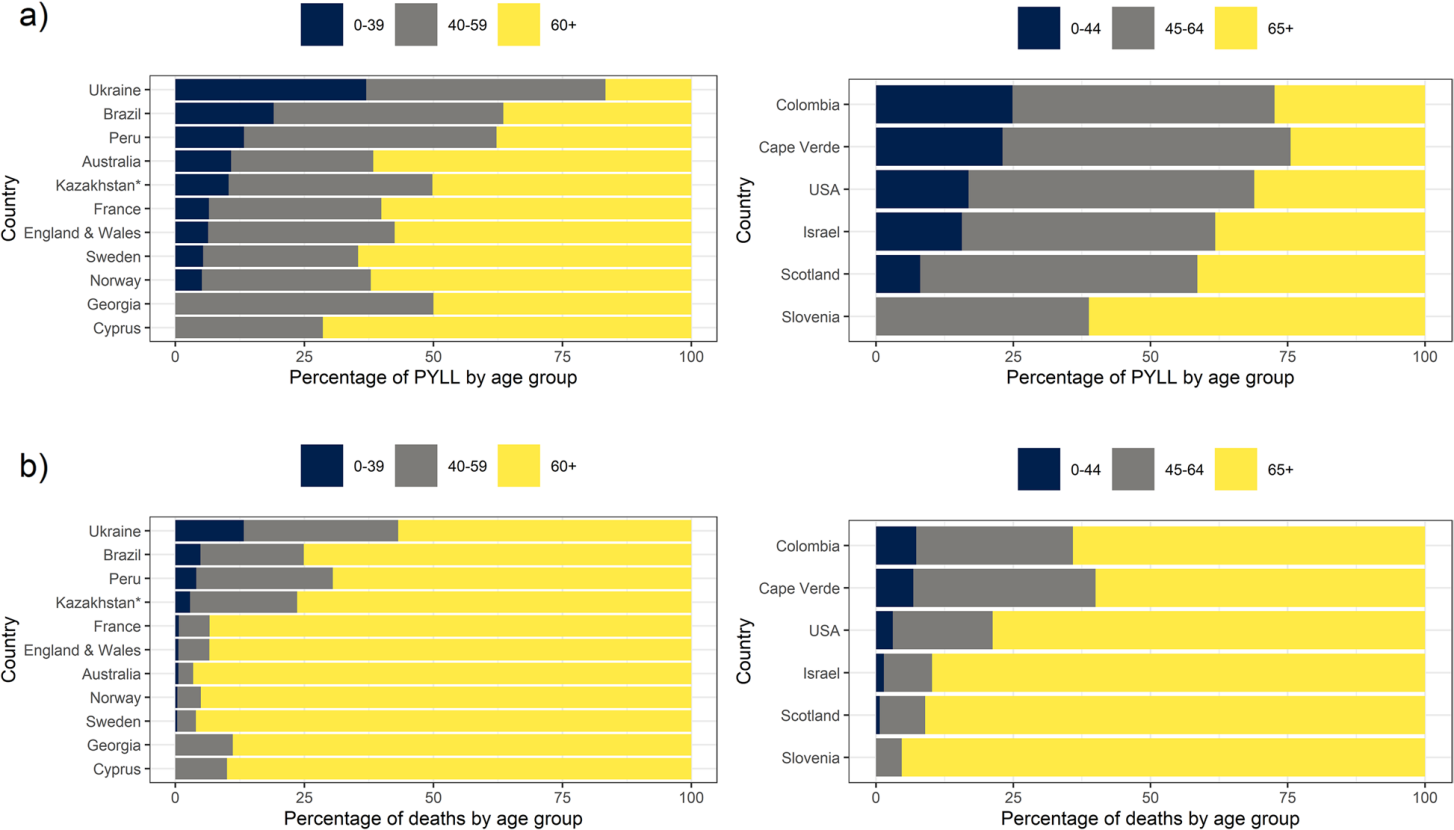
Country-specific proportions of **a)** PYLL and **b)** deaths for each of three wide age groups, chosen depending on country specific age breakdowns. Countries are presented in decreasing proportion of PYLL or deaths in the youngest age group (under 40 or 45 years of age) *For Kazakhstan data was available up to week 31, 2020

Figure 3 displays the country-specific proportions of PYLL and deaths for each of three wide age groups, chosen depending on country specific age breakdowns. The highest percentage of COVID-19 deaths was observed among the oldest age group (60+ or 65+) for all participating countries; yet the largest proportion of PYLL were attributed to the oldest age group for only some of them. Specifically, in Brazil, Peru, Ukraine, Cape Verde, Colombia, Israel, Scotland and the USA, the majority of PYLL were experienced by the middle age group (40–59 or 45–64 years).

Looking at the more detailed age breakdowns of each country (Table 1), we observed gender differences in COVID-19 number of deaths and PYLL across age groups for a few countries only. In Cyprus, England & Wales, and Norway, males reported the largest number of deaths and PYLL in a younger age group compared to females. In addition, in Kazakhstan, Peru, and Sweden, PYLL were also concentrated in the younger male age groups. In contrast, this trend was reversed for Georgia, with the most PYLL in a younger female cohort, compared to males. Overall, the largest number of PYLL was observed in younger age groups compared to the age groups with the largest number of deaths, with the exception of Kazakhstan (Table 1).

**Table 1:** Age-groups with the highest number of deaths and PYLL due to COVID-19 in each of the countries, overall and by sex, up to the end of week 35 (2020)

	DEATHS			PYLL		
Country	*Males*	*Females*	*Total*	*Males*	*Females*	*Total*
Australia	80–89	80–89	80–89	70–79	70–79	70–79
Brazil	70–79	70–79	70–79	60–69	60–69	60–69
Cape Verde	65–79	65–79	65–79	50–64	50–64	50–64
Colombia	65–79	65–79	65–79	45–64	45–64	45–64
Cyprus	60–69*	70–79	70–79	60–69*	70–79	60–69
England & Wales	80–84*	90+	90+	60–64*	70–74	60–64
France	80–89	80–89	80–89	60–69	60–69	60–69
Georgia	80–84	80–84	80–84	65–69	45–49**	45–49
Israel	80+	80+	80+	65–74	65–74	65–74
Kazakhstan^a^	60–69	60–69	60–69	50–59*	60–69	60–69
Norway	80–89*	90–99	80–89	60–69*	70–79	60–69
Peru	65–69	65–69	65–69	55–59*	60–64	55–59
Scotland	80+	80+	80+	45–64	45–64	45–64
Slovenia	80+	80+	80+	65–74	65–74	65–74
Sweden	80–89	80–89	80–89	60–69*	70–79	60–69
Ukraine	80+	80+	80+	40–54	40–54	40–54
USA	80+	80+	80+	55–64	55–64	55–64

^a^Data only available to the end of week 31, 2020

*The majority of deaths and/or PYLLs among males were observed in a younger age group compared to females

**The majority of deaths and/or PYLLs among females were observed in a younger age group compared to males

### PYLL attributed to COVID-19 by sex

**Fig. 4:**
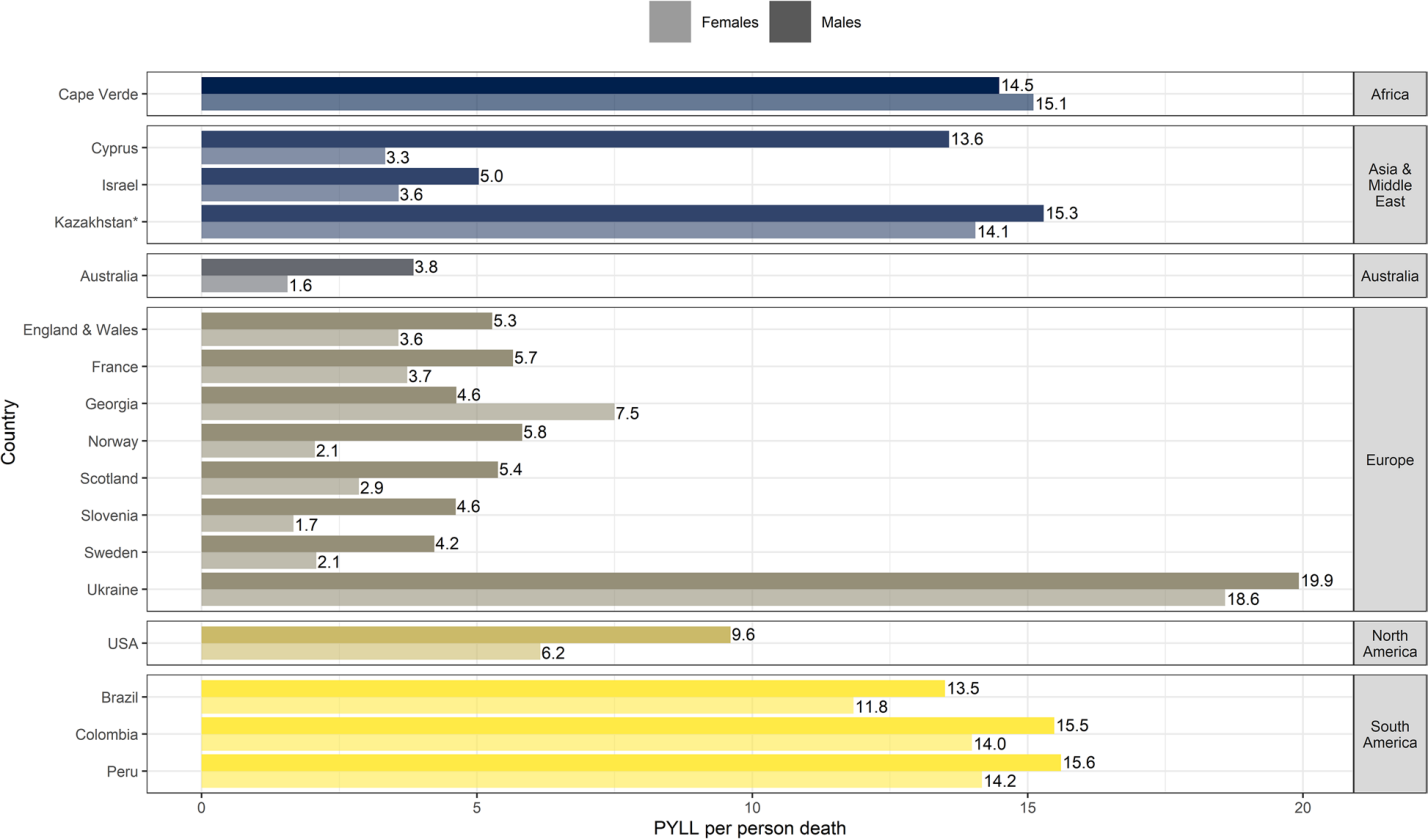
PYLL per person death by sex and country *For Kazakhstan data was available up to week 31, 2020

**Fig. 5:**
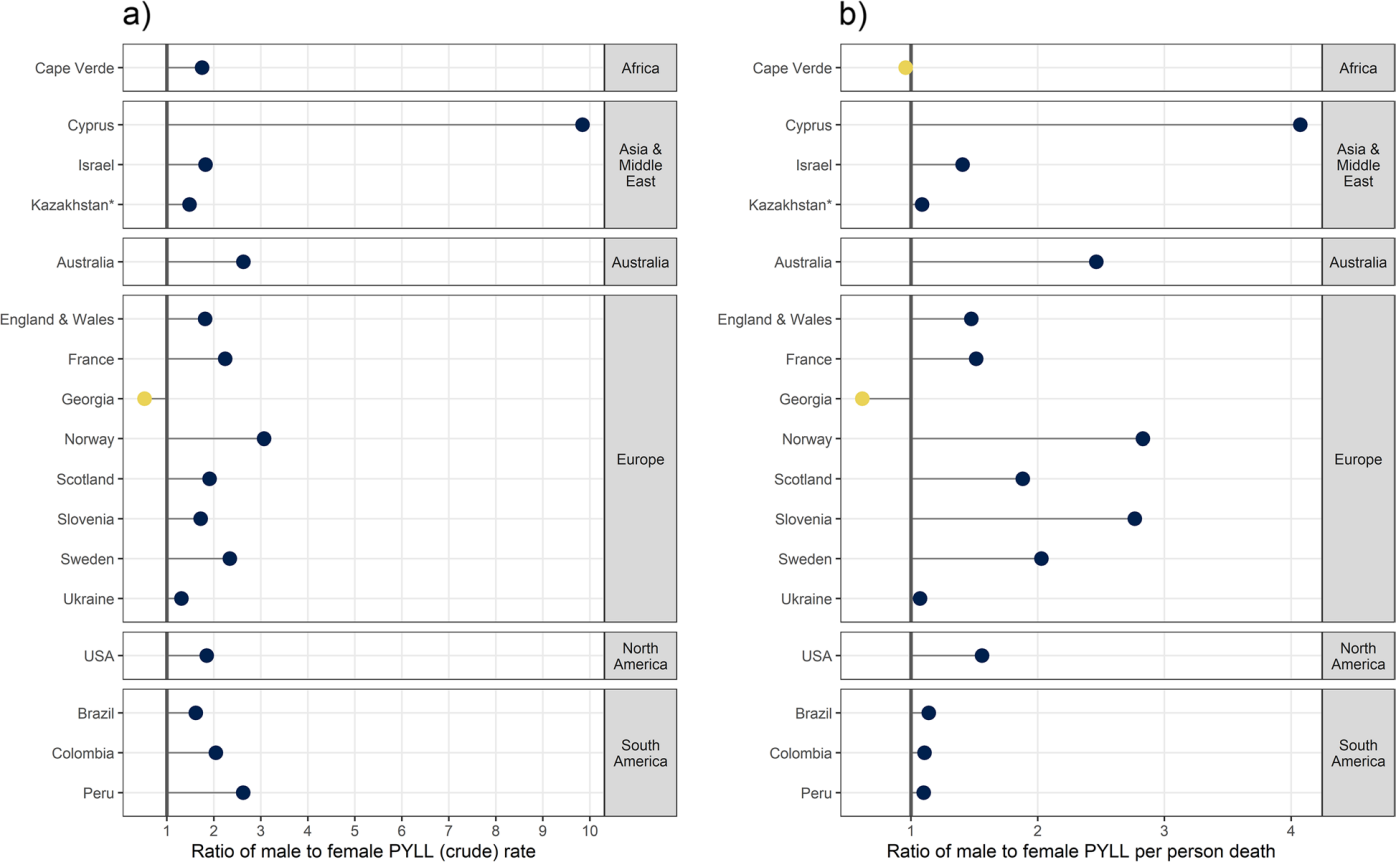
Ratio of male to female **a)** PYLL rates and **b)** PYLL per person deaths. Countries where genders were equally affected have ratios closer to the vertical line at 1, while countries where males were more severely affected display points lying to the right of the vertical line

With a total of 4,210,654.14 PYLL attributed to COVID-19 in 17 countries, 64.3% were accumulated in males and 35.7% in females. In all countries, besides Georgia, the impact of COVID-19 on PYLL was more pronounced in males compared to females (Figs. 4 and 5). While most countries displayed a small difference (1.2–2.5 PYLL per person death) between the two sexes, Cyprus, Norway, and Slovenia presented more pronounced differences (PYLL per death male to female ratio > 2).

PYLL per person death demonstrated the same sex-specific pattern as PYLL rates, except for Cape Verde, Kazakhstan, Ukraine, Colombia, and Peru where the per death male to female ratio was close to 1.

### PYLL per COVID-19 death definition

As expected, more inclusive definitions of a COVID-19 death impacted our findings. Those countries reporting COVID-19 deaths either as those certified as COD or with the disease as a contributing factor presented three times the mean PYLL rate as compared to those where only as COD is allowed. (Supplementary Table S3).

### PYLL rate and excess mortality rate

**Fig. 6:**
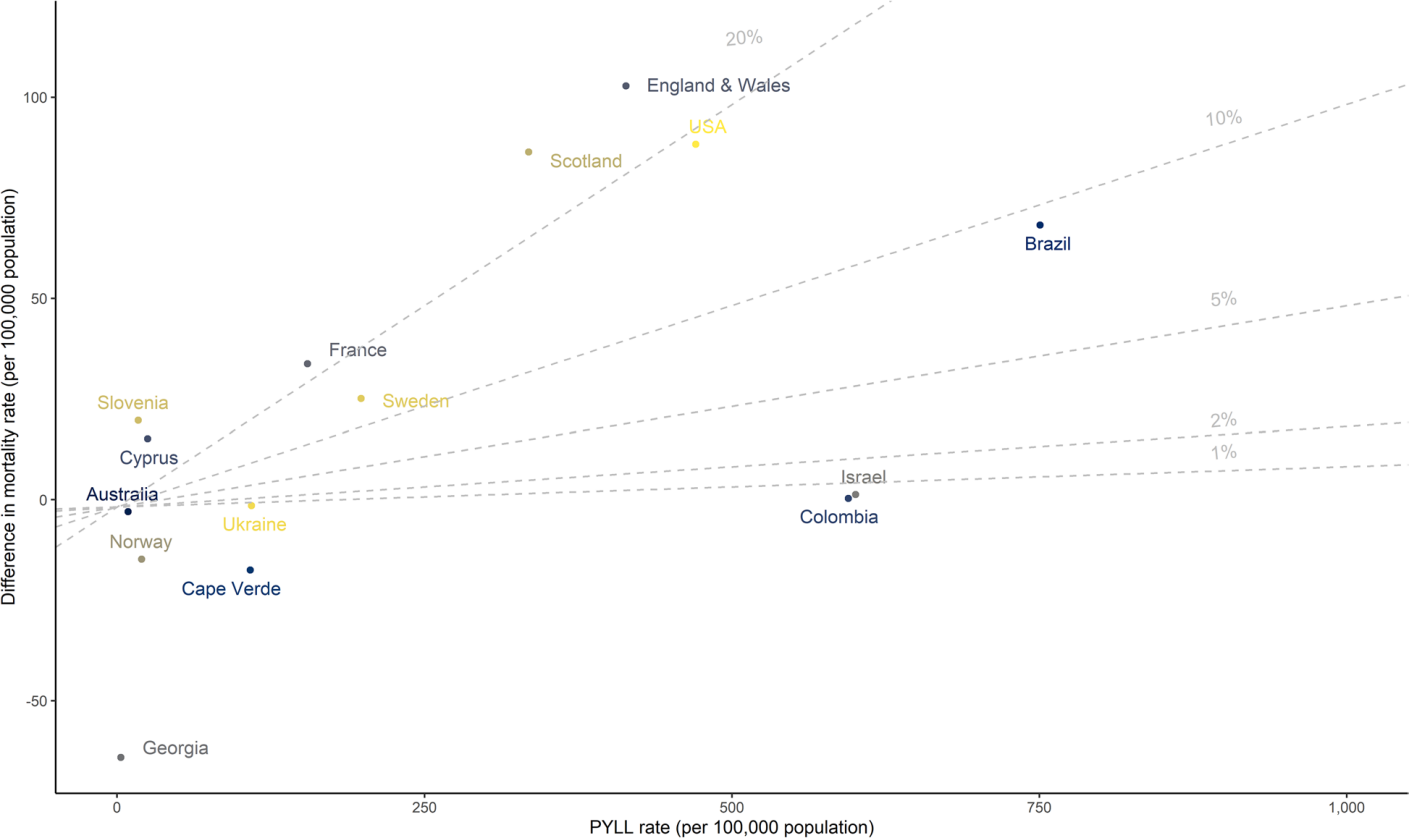
Scatterplot of the difference in mortality (2020 versus 2015–2019, per 100,000 population) versus PYLL rate (per 100,000 population). The grey lines show the ratio between difference in mortality and PYLL rate in percentage. (Note: Kazakhstan and Peru are not displayed)

Countries that showed high PYLL rates (England & Wales, Scotland, the USA, Brazil, Colombia, and Israel), also experienced (except for Colombia and Israel) excess mortality per 100,000 during the study period in 2020 compared to the mortality of the previous five years (2015–2019) (Fig. 6). Kazakhstan and Peru are not displayed because, due to data limitations, excess mortality could not be estimated.

## Discussion

The aim of this study was to assess COVID-19 impact on premature mortality using PYLL estimates from 17 countries and territories around the world from January to August 2020. Our analysis delivers three main key findings. First, the results evidenced COVID-19 as a cause of premature mortality in all the countries included in this study, with some countries being significantly more affected than others. Second, the largest proportion of PYLL was observed among the oldest (60+ or 65+) or the middle age group (40–59 or 45–64 years), depending on the country. Third, males died on average two years younger than females.

### PYLL per person death

The estimates of the number of PYLL per death indicate that those dying due to COVID-19 in European countries, Israel and Australia were on average older than those who died due to COVID-19 in South America, Ukraine, Cyprus, Cape Verde, and Kazakhstan.

In our study, the average PYLL per person death was 8.7 years, which is lower than in other studies by several years. Arolas et al. (2020) estimated on average 14.5 years of life lost (YLL) per death in 42 countries [7]; Hanlon et al. (2021) computed 14 YLL in men and 12 YLL in women in UK per COVID-19 death [22]; and Elledge et al. (2020) counted 13.25 person-years death due to COVID-19 in the USA [23]. Mitra et al. (2020), who used a similar methodology to that used in this paper but for shorter time period (as of 30 May 2020), estimated 5.5 PYLL per person death in the USA [6] which is 2.4 years less than our estimate for USA (7.9 years).

The discrepancies between our results and some of the studies mentioned above, is likely explained by differences in methodology. Most of the above-mentioned studies used as the upper age limit the Japanese female’s life expectancy of 87 years as of 2019 (the highest life expectancy in the world) [24], WHO life tables or national life tables (which attributes YLL to all ages no matter the age of death) [6]. Using a higher life expectancy allows the inclusion of deaths in those older than 80 years in the YLL calculation, and thus leads to more YLL in countries with more deaths after the age of 80, while keeping constant the YLLs of countries with lower life expectancies (where most deaths would have happened before 80 years). This also explains why these publications used the YLL notation, rather than the more conservative PYLL term. Moreover, for the UK countries, where the PYLL per person death observed in this study were half of what was elsewhere reported [22], it was previously shown that accounting for prevalent comorbid conditions in the population when estimating PYLL, substantially reduces the PYLL estimates [22].

### PYLL per 100,000 population

Europe showed the greatest differences in PYLL rates among the countries included in its geographical territory. Europe included some countries with a high number of PYLL per 100,000 such as England, Wales, and Scotland (numbers comparable to the PYLL in North America), as well as countries with very few PYLL per 100,000 population like Georgia, Slovenia, and Norway. This variability in PYLL among European countries follows the variable excess mortality experience of the European countries observed [9, 25–30].

Among South American countries, Peru had the greatest amount of PYLL per 100,000 population followed by Brazil and Colombia. A previous study, attributed the high burden of the COVID -19 disease in South America to the underlying social inequalities and to the public health limited capacities in the area [31].

In Europe, England & Wales, Sweden, and Scotland suffered more PYLL per 100,000 population than the rest of the European countries, supporting COVID-19’s impact on premature mortality. Interestingly, these countries were previously shown to have suffered excess mortality due to the COVID-19 pandemic, explaining in part the high number of PYLL per 100,000 population [9, 25–30].

In African, Asian, and Middle Eastern countries and in Australia, the PYLL per 100,000 population observed were relatively low. Cape Verde, the only African country participating in this study, experienced a different timing of the COVID-19 pandemic since they started seeing a surge in cases over the summer, but the peak of the pandemic was experienced in September–October 2020 [32]. Possible under reporting [33], seasonality patterns, possible pre-existing immunity to the virus [34], and the relatively young population may all have contributed to this low PYLL estimate [35]. To explain the low PYLL estimates in the Asian and Middle Eastern countries, similar hypotheses have been studied, including the environmental factor, possible resistance of East Asians to the coronavirus due to a gene mutation, cross-immunity, but also the early public-health measures taken by their authorities and the cautious behaviours taken by their populations [36, 37]. Lastly, Australia experienced a small peak in coronavirus cases in March, but the number of cases substantially escalated in July 2020, hence the low burden in terms of PYLL up to the end of August 2020 [38].

In July 2020, Oh et al. (2020), ranked the countries by the highest amount of PYLL per 100,000 population. They observed that Belgium, United Kingdom, Italy, Sweden, France and Spain had higher PYLL per 100,000 population than the USA and the other South American countries included in this study [18]. These results are not surprising, as by July, the European countries included in the Oh et al. study were some of the most affected countries in the world whereas, at the same time, in South America the pandemic had not reached its peak [39]. On the other hand, previously in May, Mitra et al. (2020) observed more PYLL in the USA than in Germany or Italy [6]. These results demonstrate that any comparisons between study results need to be interpreted with caution as the timing of observation of each investigation can heavily influence the results.

Our methodology (using an age limit of 80 years) also contributes to fewer PYLL than truly observed in countries with higher life expectancies (like most of European countries, Middle East, and Australia), resulting in the accumulation of fewer PYLL in these countries compared to countries with lower life expectancies (like the South American and African countries in this investigation).

### Age-group differences in the number of deaths vs. PYLL

This study highlighted differences in the number of deaths versus the number of PYLL in the different age groups in the participating countries. As expected, the largest number of deaths was registered in the oldest age groups in all countries, whilst several countries observed the majority of PYLL in the middle age group (40–59 or 45–64 years). Similar results have been observed in other studies [6, 8]. Interestingly, in Peru and in Kazakhstan, most COVID-19 deaths happened in ages younger than 70 years. Possible incomplete data, underreporting in the elderly, lack of diagnostic test resources, and low proportion of elderly living in elder-care facilities (places with concentrate vulnerable people in conditions favourable to the spread of the virus) are potential explanations of this finding.

Across countries, a variable proportion of PYLL is observed for each age interval. At the same time there are large differences in the relative contribution of each age group to total PYLL, within countries which does not follow the sociodemographic index gradient observed in other studies [7].

### Sex differences in the number of deaths and PYLL

According to the data used in this study, the largest number of deaths and PYLL happened in the same age groups in most countries, irrespectively of sex.

The comparison of the PYLL results by sex highlighted the increased burden of COVID-19 in terms of premature mortality in males rather than females. Looking at all countries together, 64.3% of the total PYLL were estimated among males and 35.7% among females. In all the countries except for Georgia, males suffered more PYLL per 100,000 deaths than females.

The greater burden of COVID-19 in terms of premature mortality in males rather than females, is also highlighted by the PYLL per person death results. On average males lost 9.5 PYLL per person death whereas females lost 7.4 years. The finding that COVID-19 premature mortality seems to be heightened in males than in females has also been observed in other studies [40, 41]. Biological reasons such as the immunological response between the two sexes could partly explain this difference [42, 43]. Channappanavar et al. (2017) demonstrated that oestrogen, the female sex hormone, can play a protective role by supressing the replication of SARS-CoV virus while activating the immune response [44]. Further, angiotensin-converting enzyme 2 (ACE2) is providing a protective role. ACE2 is the host receptor for SARS-CoV-2 virus, and it is also part of the renin-angiotensin system which is crucial in tissue response to viral infection [45]. The ACE2 is overexpressed in females, and increased levels of ACE2 is expected to provide greater tissue protection after viral entry, minimizing the death rates as compared to males [46]. However, other social factors such as the differential distribution of unhealthy behaviours, like smoking or alcohol consumption, as well as the increased likelihood of females to adhere to social precautions to reduce the exposure to SARS-CoV-2 might also contribute to these results [43, 47]. Lastly, given that females have a longer life expectancy than males, it is possible that the use of a common age limit may have led to an underestimation of PYLL among females. Nevertheless, further investigation in whether there is truly a gender gradient in COVID-19 premature mortality and the reasons behind it warrant further investigation [42, 48, 49].

### PYLL per COVID-19 death definition

In this study, it was also observed that countries who reported as COVID -19 deaths, deaths where COVID-19 was either a cause of death or a contributing cause to death, observed on average more PYLL per 100,000 population than countries who adopted a stricter definition of COVID-19 deaths, and only reported deaths were COVID-19 was a cause of death. This result may be indicating that individuals where COVID-19 was present but perhaps not taking part in the chain of events leading to death, died on average at a younger age, pointing to the presence of comorbidities that most likely also explained the deaths. To this extent, this finding may be testifying to an over-estimate of COVID-19 deaths associated with the classification of all deaths in SARS-COV-2 positive persons as COVID-19 specific deaths. However, this estimate could be partly driven by the adoption of more inclusive definitions of a COVID-19 death by some of the most highly impacted countries in terms of excess mortality such as Brazil and the USA [21].

It may be worth highlighting here that in our study we report the PYLL estimates for each country based on the COVID-19 definition used at the national level. Regional comparisons are not expected to be significantly biased since almost all countries within a region follow the same definition.

### PYLL rate against excess mortality during the study period

Lastly, of the countries displaying high PYLL rates in this study, all countries (except for Colombia and Israel) also experienced excess mortality per 100,000 population during the study period in 2020 compared to the mortality of the previous five years. This result suggests that excess mortality during the COVID-19 pandemic is accompanied by a large impact in terms of premature mortality.

### Strengths and limitations

Our study has some important strengths compared to other published studies. It is one of the few studies attempting a comparison of the burden of COVID-19 premature mortality across 17 countries from different regions, some of which have not been previously studied. In addition, this study explored PYLL per 100,000 population and per person death and has also explored the differential impact of age and sex on these estimates. Furthermore, the data used in this study is primarily data from national sources, which is more reliable than publicly available data, often used in other similar publications.

This study has several data and methodological limitations. We obtained data up to the end of August 2020 (week 35) for all participating counties apart from Kazakhstan, where the data was available only until the end of week 31. As a result, we might have underestimated the PYLL for Kazakhstan compared to other countries, as well as the total PYLL estimate for the whole study period. Nevertheless, subgroup comparisons are not expected to be affected by this.

Another data limitation that poses a challenge in the comparison of results between countries is that each country grouped deaths in different age groups (see Supplementary Table S2). The estimates from countries with smaller range of age groups, would have more reliability than estimates from countries that have 20 or more years range age-groups, as in the latter case, the midpoint of the age group might be farther from the real age of each death.

Other studies found a strong association between the presence of comorbidities, such as cardiovascular disease, hypertension, diabetes, congestive heart failure, chronic kidney disease and cancer, and the risk of death due to COVID-19 [50]. The lack of accessible information on the presence of comorbidities among those who died in the countries included in these studies precluded us from correcting our results for comorbidities. However, aware of this limitation, we cautiously chose the methodology for this study, opting for a more conservative method than others have used. Other methodologies, like the national life tables or the WHO tables, that account for some few YLL no matter the age of the person, or the use of the Japanese female’s life expectancy as upper age limit, that is the highest in the world would lead to higher PYLL estimates which would not be as realistic given that the life expectancy of patients with the aforementioned comorbidities is shorter than that of the general population. Results of other studies where PYLL estimates are given before and after adjustment for comorbidities, support our choice in methodology [22].

The use of a standard life expectancy also has its limitations. The upper age limit was the same for all the countries and sexes, resulting in fewer PYLL in those countries with life expectancies higher than 80 years and in females, who on average die a few years later than men. However, despite its limitations, this methodology has been strongly recommended for comparison across countries [6, 51, 52].

Lastly, given the relevance of post-acute COVID-19 syndrome [53], it would be of much interest to include in investigations of COVID-19 burden, the metric of disability as in the Disability-Adjusted Life Year (DALY indicator). Even though such data was not available to the consortium at the time of publication, DALYs would provide more holistic estimates of health burden due to COVID-19 because the indicator takes into account post-acute and chronic effects which are likely to be more relevant among adults than premature deaths. Even though some attempts to estimate DALYs due to COVID-19 have been made [54, 55],more geographically diverse investigations are warranted.

## Conclusion

In this investigation, South American countries were evidenced as the most impacted countries in terms of COVID-19 premature mortality. At the same time, countries in Asia and Middle East as well as Africa (Cape Verde), were least affected. The timing of the pandemic, seasonal trends, the control measures enforced, and underlying social conditions, together with the demographic characteristics are probable explanations for the differences observed among countries.

As the pandemic is ongoing and new coronavirus strains are appearing, different countries are adopting different strategies that are tailored to their respective social, economic, and health situation. Undoubtedly, observational studies on the excess mortality from 2020 onwards will shed more light on who is most affected in terms of COVID-19 premature mortality, but in their absence, PYLL investigations can be of immense support to policy-makers and public health decision makers as they plan and implement appropriate and proportionate public health actions.

## Supplementary Information

**Additional file 1.**

## Data Availability

The datasets analysed during the current study are included in this published article and its supplementary information files. All partners received permission to access and use the data from the corresponding national dataset source listed in Supplementary Table 1. The weekly datasets analyzed during the current study are not publicly available, but are available from the national primary source upon request.

## Abbreviations

WHO: World Health Organization
PYLL: Potential Years of Life Lost
YLL: Years of Life Lost
USA: United States
COD: cause of death
DALY: Disability-Adjusted Life Year
