# Supplementary material for: Premature mortality attributable to COVID-19: potential years of life lost in 17 countries around the world, January–August 2020
Source: BMC Public Health. 2022 Jan 9;22:54. doi: 10.1186/s12889-021-12377-1 (PMC8743065; doi:10.1186/s12889-021-12377-1)
Supplement: Supplementary file 1 — Additional file 1. [file 12889_2021_12377_MOESM1_ESM.pdf]

SUPPLEMENTARY FILE

Supplementary Table 1. Summary of life expectancy, time unit, COVID-19 deaths definition, and national data source per country.

Country	Life Expectancy***	Time unit	Type of COVID-19 Deaths reported		Source
			Cause of death	Cause of death or contributing condition	
**Australia**	83	National week	x		Australian Bureau of Statistics
**Brazil**	76	ISO		x	The surveillance system of severe acute respiratory syndromes, Ministry of Health
**Cape Verde**	73	Month		x	Ministry of Health; Integrated surveillance system and emergency response; National Public Health Institute
**Colombia**	77	Month		x	Instituto Nacional de Salud
**Cyprus**	83	ISO	x		Epidemiological Surveillance Unit, Ministry of Health
**England and Wales**	81	National week	x		Office for National Statistics
**France***	83	ISO	x		National Institute of Demographic Studies/Public Health France
**Georgia**	74	ISO	x		Vital Registration System of the National Center for Disease Control and Public Health National Statistics Office of Georgia
**Israel**	83	Epi		x	Ministry of Health
**Kazakhstan****	73	ISO	x		Ministry of Health
**Norway**	83	ISO		x	Norwegian Surveillance System for Communicable Diseases
**Peru**	77	ISO		x	Ministry of Health
**Scotland**	79	ISO	x		National Records of Scotland

**Slovenia**	81	ISO		x	National Institute of Public Health
**Sweden**	83	Month	x		National Board of Health and Welfare
**Ukraine**	72	ISO	x		Cabinet of Ministers of Ukraine
**United States**	79	Epi		x	National Center for Health Statistics

Abbreviations: ISO: International Organization for Standardization; Epi: epidemiological

*COVID-19 deaths in France only accounted for those who died in the hospital due to COVID-19.

** Data of deaths register due COVID-19 by sex and age-group in Kazakhstan, are counting just up to week 31 of 2020 (31 included).

*** Data obtained from the World bank (https://data.worldbank.org/), except for the UK nations, for which data was obtained from the Office for National Statistics (https://www.ons.gov.uk/), and for Cyprus from Eurostat (https://ec.europa.eu/eurostat/en/) to include only the population in the Republic of Cyprus government controlled-area. Life expectancy data was based on 2018 estimates and represents the average of males' and females' life expectancy.

Supplementary Table 2. Country Specific Death and number of Potential Years of Life Lost (PYLL) per age-group and sex

Australia

Age group	Midpoint	Deaths Males	PYLL Males	Deaths Females	PYLL Females	Sex unknown	Total deaths	Total PYLL
0-29	14.5	1	65	0	0	0	1	65
30-39	34.5	2	90	0	0	0	2	90
40-49	44.5	2	70	0	0	0	2	70
50-59	54.5	9	225	4	100	0	13	325
60-69	64.5	16	240	8	120	0	24	360
70-79	74.5	69	345	36	180	0	105	525
80-89	-	105	0	116	0	0	221	0
90-99	-	64	0	87	0	0	151	0
100+	-	1		5		0	6	0
Total		269	1035	256	400	0	525	1435

Brazil

Age group	Midpoint	Deaths Males	PYLL Males	Deaths Females	PYLL Females	Sex unknown	Total deaths	Total PYLL
0-9	4.5	216	16200	190	14250	0	406	30450
10-19	14.5	160	10400	163	10595	0	323	20995
20-29	24.5	697	38335	571	31405	0	1268	69740
30-39	34.5	2316	104220	1562	70290	0	3878	174510
40-49	44.5	5400	189000	3046	106610	0	8446	295610
50-59	54.5	9985	249625	5878	146950	0	15863	396575
60-69	64.5	16804	252060	10627	159405	0	27431	411465
70-79	74.5	18307	91535	12669	63345	0	30976	154880

80-89	-	12892	0	11565	0	0	24457	0
90-99	-	3318	0	4235	0	0	7553	0
100-110	-	168	0	242	0	0	410	0
110+	-	6	0	8	0	0	14	0
Unknown	-	212	0	176	0	18	406	0
Total		70481	951375	50932	602850	18	121431	1554225

Cape-Verde

Age group	Midpoint	Males	PYLL Males	Females	PYLL Females	Sex unknown	Total deaths	Total PYLL
0-4	2	0	0	0	0	0	0	0
5-9	7	0	0	0	0	0	0	0
10-14	12	0	0	0	0	0	0	0
15-44	29.5	1.8	90	0.91	45.5	0	2.71	135.5
45-49	47	0.2	6.6	0.09	2.97		0.29	9.57
50-64	57	8	184	5	115	0	13	299
65-79	72	12	96	6	48	0	18	144
80+	-	4	0	2	0	0	6	0
Total		26	376.6	14	211.47	0	40	588.07

Colombia

Age group	Midpoint	Deaths Males	PYLL Males	Deaths Females	PYLL Females	Sex unknown	Total deaths	Total PYLL
0-4	2	10	780	11	858	0	21	1638
05.-14	9.5	12	840	7	490	0	19	1330
15-44	29.5	944	47200	459	22950	0	1403	70150
45-64	54.5	3838	95950	1772	44300	0	5610	140250
65-79	72	6331.77	50654.16	3738.54	28039.05	0	10070.31	80562.48
80+	-	1485.23	0	1054.46	0	0	2539.69	0

Total		12621	195424.16	7042	98506.32	0	19663	293930.48

Cyprus

Age group	Midpoint	Deaths Males	PYLL Males	Deaths Females	PYLL Females	Sex unknown	Total deaths	Total PYLL
0-39	19.5	0	0	0	0	0	0	0
40-49	44.5	1	35	0	0	0	1	35
50-59	54.5	1	25	0	0	0	1	25
60-69	64.5	7	105	0	0	0	7	105
70-79	74.5	5	25	4	20	0	9	45
80+	-	0	0	2	0	0	2	0
Total		14	190	6	20	0	20	210

England & Wales

Age group	Midpoint	Males	PYLL Males	Females	PYLL Females	Sex unknown	Total deaths	Total PYLL
<1	0.5	2	158	0	0	0	2	158
1-4	2.5	0	0	1	77	0	1	77
5-9	7	0	0	1	73	0	1	73
10-14	12	2	136	1	68	0	3	204
15-19	17	5	315	4	252	0	9	567
20-24	22	15	870	10	580	0	25	1450
25-29	27	32	1696	17	901	0	49	2597
30-34	32	47	2256	35	1680	0	82	3936
35-39	37	76	3268	57	2451	0	133	5719
40-44	42	164	6232	94	3572	0	258	9804
45-49	47	293	9669	184	6072	0	477	15741
50-54	52	558	15624	316	8848	0	874	24472
55-59	57	1009	23207	485	11155	0	1494	34362

60-64	62	1400	25200	717	12906	0	2117	38106
65-69	67	1877	24401	1006	13078	0	2883	37479
70-74	72	3061	24488	1699	13592	0	4760	38080
75-79	77	4245	12735	2640	7920	0	6885	20655
80-84	-	5679	0	4175	0	0	9854	0
85-89	-	5461	0	5121	0	0	10582	0
90+	-	4540	0	6711	0	0	11251	0
Total		28466	150255	23274	83225	0	51740	233480

France

Age group	Midpoint	Deaths Males	PYLL Males	Deaths Females	PYLL Females	Sex unknown	Total deaths	Total PYLL
0-9	4.5	2	150	1	75	0	3	225
10-19	14.5	2	130	2	130	0	4	260
20-29	24.5	17	935	9	495	0	26	1430
30-39	34.5	59	2655	39	1755	0	98	4410
40-49	44.5	168	5880	90	3150	0	258	9030
50-59	54.5	639	15975	302	7550	0	941	23525
60-69	64.5	1703	25545	705	10575	0	2408	36120
70-79	74.5	3087	15435	1399	6995	0	4486	22430
80-89	-	4224	0	3151	0	0	7375	0
90-120	-	1813	0	2497	0	0	4310	0
Unknown	-	78		33		137	248	0
Total		11792	66705	8228	30725	137	20157	97430

Kazakhstan

Age group	Midpoint	Deaths Males	PYLL Males	Deaths Females	PYLL Females	Sex unknown	Total deaths	Total PYLL
0-9	4.5	0	0	0	0	0	0	0

10.-19	14.5	0	0	0	0	0	0	0
20-29	24.5	1	55	7	385	0	8	440
30-39	34.5	9	405	10	450	0	19	855
40-49	44.5	49	1715	27	945	0	76	2660
50-59	54.5	125	3125	73	1825	0	198	4950
60-69	64.5	191	2865	141	2115	0	332	4980
70-79	74.5	147	735	115	575	0	262	1310
80-89	-	57	0	65	0	0	122	0
90+	-	3	0	10	0	0	13	0
Unknown	-	0	0	0	0	0	0	0
Total		582	8900	448	6295	0	1030	15195

Georgia

Age group	Midpoint	Deaths Males	PYLL Males	Deaths Females	PYLL Females	Sex unknown	Total deaths	Total PYLL
0-4	2	0	0	0	0	0	0	0
5.-9	7	0	0	0	0	0	0	0
10.-14	12	0	0	0	0	0	0	0
15-19	17	0	0	0	0	0	0	0
20-24	22	0	0	0	0	0	0	0
25-29	27	0	0	0	0	0	0	0
30-34	32	0	0	0	0	0	0	0
35-39	37	0	0	0	0	0	0	0
40-44	42	0	0	0	0	0	0	0
45-49	47	0	0	1	33	0	1	33
50-54	52	0	0	0	0	0	0	0
55-59	57	0	0	1	23	0	1	23
60-64	62	0	0	0	0	0	0	0
65-69	67	2	26	0	0	0	2	26
70-74	72	1	8	2	16	0	3	24

75-79	77	1	3	1	3	0	2	6
80-84	82	4	0	3	0	0	7	0
85-89	-	0	0	2	0	0	2	0
90+	-	0	0	0	0	0	0	0
Total		8	37	10	75	0	18	112

Israel

Age group	Midpoint	Deaths Males	PYLL Males	Deaths Females	PYLL Females	Sex unknown	Total deaths	Total PYLL
0-4	2	0	0	0	0	0	0	0
05.-14	9.5	0	0	0	0	0	0	0
15-24	19.5	1	60	2	120	0	3	180
25-34	29.5	2	100	2	100	0	4	200
35-44	39.5	4	160	2	80	0	6	240
45-54	49.5	11	330	12	360	0	23	690
55-64	59.5	38	760	19	380	0	57	1140
65-74	69.5	95	950	42	420	0	137	1370
75-79	77.5	40.5	81	31.93	63.86	0	265	144.86
80+	-	293.5		315.07			416	
Total		485	2441	426	1523.86	0	911	3964.86

Norway

Age group	Midpoint	Deaths Males	PYLL Males	Deaths Females	PYLL Females	Sex unknown	Total deaths	Total PYLL
0-9	4.5	0	0	0	0	0	0	0
10.-19	14.5	0	0	0	0	0	0	0
20-29	24.5	1	55	0	0	0	1	55
30-39	34.5	0	0	0	0	0	0	0
40-49	44.5	3	105	2	70	0	5	175

50-59	54.5	7	175	0	0	0	7	175
60-69	64.5	19	285	5	75	0	24	360
70-79	74.5	39	195	22	110	0	61	305
80-89	84.5	47	0	45	0	0	92	0
90-99	94.5	24	0	48	0	0	72	0
≥100		0	0	2	0	0	2	0
Total		140	815	124	255	0	264	1070

Peru

Age group	Midpoint	Deaths Males	PYLL Males	Deaths Females	PYLL Females	Sex unknown	Total deaths	Total PYLL
0-4	2	23	1794	22	1716	0	45	3510
5.-9	7	18	1314	12	876	0	30	2190
10.-14	12	13	884	12	816	0	25	1700
15-19	17	22	1386	14	882	0	36	2268
20-24	22	57	3306	38	2204	0	95	5510
25-29	27	92	4876	42	2226	0	134	7102
30-34	32	207	9936	113	5424	0	320	15360
35-39	37	364	15652	125	5375	0	489	21027
40-44	42	647	24586	226	8588	0	873	33174
45-49	47	1123	37059	366	12078	0	1489	49137
50-54	52	1674	46872	504	14112	0	2178	60984
55-59	57	2360	54280	796	18308	0	3156	72588
60-64	62	2805	50490	1121	20178	0	3926	70668
65-69	67	2931	38103	1299	16887	0	4230	54990
70-74	72	2708	21664	1211	9688	0	3919	31352
75-79	77	2181	6543	1018	3054	0	3199	9597
80-84	-	1669	-	845	-	0	2514	-
85-89	-	1032	-	551	-	0	1583	-
90-94	-	408	-	248	-	0	656	-
95-99	-	83	-	72	-	0	155	-

100-104	-	10	-	5	-	0	15	-
104-109	-	1	-	0	-	0	1	-
Total		20428	318745	8640	122412	0	29068	441157

Scotland

Age group	Midpoint	Males	PYLL Males	Females	PYLL Females	Sex unknown	Total deaths	Total PYLL
<1	0.5	0	0	0	0	0	0	0
1.-14	7.5	0	0	0	0	0	0	0
15-44	29.5	14	700	14	700	0	28	1400
45-64	54.5	230	5750	121	3025	0	351	8775
65-74	69.5	375	3750	229	2290	0	604	6040
75-79	77	363.78	1091.34	25.6	76.8	0	389.38	1168.14
80+	-	1115.22	0	1741.4	0	0	2856.62	0
Total		2098	11291.34	2131	6091.8	0	4229	17383.14

Slovenia

Age group	Midpoint	Males	PYLL Males	Females	PYLL Females	Sex unknown	Total deaths	Total PYLL
0-14	7	0	0	0	0	0	0	0
15-44	29.5	0	0	0	0	0	0	0
45-54	49.5	1	30	1	30	0	2	60
55-64	59.5	3	60	1	20	0	4	80
65-74	69.5	10	100	5	50	0	15	150
75-79	77	13.6	40.8	10.05	30.15	0	23.65	70.95
80+	-	22.4		60.95		0	83.35	0
Total		50	230.8	78	130.15	0	128	360.95

Sweden

Age group	Midpoint	Deaths Males	PYLL Males	Deaths Females	PYLL Females	Sex unknown	Total deaths	Total PYLL
0-9	4.5	0	0	0	0	0	0	0
10-19	14.5	0	0	1	65	0	1	65
20-29	24.5	4	220	3	165	0	7	385
30-39	34.5	8	360	5	225	0	13	585
40-49	44.5	32	1120	14	490	0	46	1610
50-59	54.5	134	3350	34	850	0	168	4200
60-69	64.5	305	4575	112	1680	0	417	6255
70-79	74.5	806	4030	442	2210	0	1248	6240
80-89	-	1319	0	1178	0	0	2497	0
90+	-	622	0	939	0	0	1561	0
Total		3230	13655	2728	5685	0	5958	19340

Ukraine

Age group	Midpoint	Deaths Males	PYLL Males	Deaths Females	PYLL Females	Sex unknown	Total deaths	Total PYLL
0-19	9.5	39	2730	21	1470	0	60	4200
20-39	29.5	142	7100	118	5900	0	260	13000
40-54	47	249	8217	247	8151	0	496	16368
55-59	57	122.85	2825.55	102.98	2368.54	0	225.83	5194.09
60-69	64.5	228.15	3422.25	168.02	2520.3	1	397.17	5942.55
70-79	74.5	157.74	788.7	203.36	1016.8	0	361.1	1805.5
80+	-	320.26		292.64			612.9	
Total		1259	25083.5	1153	21426.64	1	2413	46510.14

United States

Age group	Midpoint	Deaths Males	PYLL Males	Deaths Females	PYLL Females	Sex unknown	Total deaths	Total PYLL
0-4	2	21	1638	15	1170	0	36	2808

5.-14	9.5	22	1540	10	700	0	32	2240
15-24	19.5	216	12960	130	7800	0	346	20760
25-34	29.5	963	48150	489	24450	0	1452	72600
35-44	39.5	2597	103880	1196	47840	0	3793	151720
45-54	49.5	6803	204090	3164	94920	0	9967	299010
55-64	59.5	15374	307480	8299	165980	0	23673	473460
65-74	69.5	24552	245520	15353	153530	0	39905	399050
75-79	77	12077.1	36231.3	8794.4	26383.2	0	20871.5	62614.5
80+	-	37483.9	0	47523.6	0	0	85007.5	0
Total		100109	961489.3	84974	522773.2	0	185083	1484262.5

Supplementary Table 3. Potential Years of Life Lost (PYLL) by type of COVID-19 death reported

	Country	PYLL per 100.000
COVID-19 as Cause of death	Australia	8.9
	Cyprus	24.9
	Kazakhstan	83.4
	England & Wales	413.9
	France	154.8
	Georgia	3.1
	Scotland	334.7
	Sweden	198.4
	Ukraine	109.4
	Mean	147.9
COVID-19 as Cause of death or contributing condition	Brazil	750.6
	Colombia	594.7
	Peru	1381.0
	United States	470.7
	Cape Verde	108.3
	Slovenia	17.3
	Norway	20.0
	Israel	600.7
	Mean	492.9

Africa
Asia & Middle East
Australia
Europe
North America
South America
